# Ebola and Marburg Hemorrhagic Fevers: Neglected Tropical Diseases?

**DOI:** 10.1371/journal.pntd.0001546

**Published:** 2012-06-26

**Authors:** Adam MacNeil, Pierre E. Rollin

**Affiliations:** Viral Special Pathogens Branch, the Centers for Disease Control and Prevention, Atlanta, Georgia, United States of America; University of Texas Medical Branch, United States of America

## Abstract

Ebola hemorrhagic fever (EHF) and Marburg hemorrhagic fever (MHF) are rare viral diseases, endemic to central Africa. The overall burden of EHF and MHF is small in comparison to the more common protozoan, helminth, and bacterial diseases typically referred to as neglected tropical diseases (NTDs). However, EHF and MHF outbreaks typically occur in resource-limited settings, and many aspects of these outbreaks are a direct consequence of impoverished conditions. We will discuss aspects of EHF and MHF disease, in comparison to the “classic” NTDs, and examine potential ways forward in the prevention and control of EHF and MHF in sub-Saharan Africa, as well as examine the potential for application of novel vaccines or antiviral drugs for prevention or control of EHF and MHF among populations at highest risk for disease.

## Introduction

Ebola hemorrhagic fever (EHF) and Marburg hemorrhagic fever (MHF) are two similar clinical diseases caused by viruses of the genera *Ebolavirus* (EBOV) and *Marburgvirus* (MARV), respectively, both of the family Filoviridae [1]. Owing to largely sensationalist accounts of outbreaks [2], these diseases are widely recognized, despite the overall rarity of their occurrence [3]. However, EBOV and MARV are highly pathogenic, and have traditionally been associated with devastating outbreaks, with case fatality ranging from 25% to 90% [4]. Additionally, EBOV and MARV are considered potential bioweapons agents [5], and as such are classified as class A select agents. While the ability to conduct research on infectious EBOV and MARV is limited to a small number of high containment laboratories, extensive funding has been applied to primary research in the past decade, and progress has been made in understanding the biology of these viruses, as well as toward development of potential therapies [6], [7]. However, from the perspective of those at most risk of disease, this progress has not been experienced. Large outbreaks of EHF in the Democratic Republic of Congo (DRC) in 2007 and 2008, and in Uganda in 2007 [8]–[10], have demonstrated the continued potential for prolonged virus transmission in impoverished rural communities. We will discuss the epidemiology and control of EHF and MHF, relative to the concepts of neglected tropical diseases (NTDs).

## Epidemiology of Ebola and Marburg Hemorrhagic Fever

EBOV and MARV are zoonotic viruses, and outside of outbreaks, do not persist in human populations. Current data suggest fruit bats as the reservoir of EBOV and MARV, and the distribution of both viruses appears to be limited to sub-Saharan Africa [11]–[14] (with the exception of *Reston ebolavirus* (REBOV), identified in the Philippines, and not recognized to be associated with human disease [15], [16]). Clusters and outbreaks are primarily the result of person-to-person transmission of these viruses, which occurs through direct contact with the body, bodily fluids (commonly to health care workers), or contaminated clothes or linens of an infected person [17]–[21]. The level of viremia, and thus presumptively the risk of transmission, corresponds with disease severity, with highest concentrations of the virus during later stages of disease [22], [23].

Three distinct contact modalities account for virus transmission during outbreaks (summarized in Table 1): 1) transmission between family members, close contacts, and care givers of sick individuals; 2) contact with dead bodies during preparation and funeral proceedings; and 3) transmission in health care settings from sick patients to medical staff or to other hospitalized patients by breaches in barrier nursing and reusing medical equipment [18], [19], [24]–[28]. As a result, outbreak response involves three major components: 1) daily observation of all contacts of sick individuals, so that upon onset of illness, persons can be transported to medical facilities and avoid further transmission in the community; 2) ensuring safe burials of deceased individuals; and 3) establishment of patient isolation wards, with medical staff equipped with and trained in usage of personal-protective equipment, to block health care–associated transmission of the virus [26], [29]–[34].

**Table 1:** Common routes of EHF and MHF spread, and interventions to stop transmission during outbreaks.

Route of Spread	Intervention
Community transmission to family members and other close contacts of EHF or MHF cases	Daily monitoring of all contacts of EHF and MHF cases and rapid transfer of sick contacts to medical facility for evaluation
Contact with deceased EHF or MHF cases during preparation of the body or funeral proceedings	Implementation of safe burial practices for all deceased individuals
Transmission in the health care setting from EHF or MHF cases to medical staff by direct contact or contact with bodily fluids, or to other patients through contaminated medical equipment	Establishment of isolation ward and provide clinical care by medical staff with training specific to EHF and MHF outbreaks

While logistically challenging, the above interventions are not technologically difficult. These have consistently been applied in outbreaks, and are effective in stopping the chains of transmission. So why do large EHF and MHF outbreaks continue to occur? Response activities are contingent on identification of the outbreak. A common occurrence among large outbreaks is the large lag, often in the range of months, between initial cases and actual detection of EBOV or MARV [35]. Typical symptoms of EHF and MHF, such as fever, vomiting, diarrhea, fatigue, headache, and myalgia [9], [24], [25], [36], [37], can be mistaken for other more frequent endemic tropical infections. However, the fact that outbreaks occur most commonly in resource-limited settings should not be overlooked. Other important aspects include limited capacity of medical and public health staff to conduct disease surveillance and the inability to rapidly perform diagnostic testing.

Additionally, while the zoonotic source of exposure is not always identified in outbreaks, introductions of these viruses to human populations have been associated with entering caves and mines (for MARV) and hunting for or processing bushmeat (for EBOV) [38]–[45]. Educational interventions aimed at discouraging these activities (or potentially directly blocking physical access to caves or mines) have the potential to limit introduction of EBOV and MARV into human populations. For instance, education outreach was performed in the border region of Republic of the Congo (RoC) and Gabon after a series of EHF outbreaks occurred over numerous years, starting in 1994; however, no outbreaks have occurred since 2005 [44]. Similarly, introduction of MARV occurred for numerous years among miners in Watsa Zone of DRC [39], and cases of MHF ceased only following flooding of the mine [46]. Finally, among routes of filovirus transmission to humans, it may be important to consider the role of other potential secondary hosts. REBOV, and its association with primates from the Philippines, was identified previously [47]. While serologic evidence indicated that humans exposed to infectious primates may be infected, REBOV does not appear to cause overt disease in humans [47], [48]. Interestingly, REBOV was recently identified in commercial swine in the Philippines, and similarly, evidence of seropositive humans exposed to these animals was observed [15]. Recent laboratory studies have demonstrated that REBOV, as well as ZEBOV, not only infects, but also may be transmitted among swine [49], [50]. The scenario that either a pathogenic filovirus may enter (and be transmitted among swine) or that mutations in REBOV may result in a virus pathogenic to humans should continue to be considered in global surveillance efforts. In addition to the direct impact on human health, the potential economic impact on agricultural production, if swine (or other livestock) are a direct or perceived threat for transmission of filoviruses, would likely be devastating to a local or regional economy.

## Ebola and Marburg Hemorrhagic Fever as NTDs

Currently there is no standardized definition of an NTD [51], [52], and various groups have applied differing standards in the classification of NTDs. Liese et al. summarized two district approaches to characterizing NTDs, the first as “neglect as the defining characteristic”, and the second as “the diseases' shared features and their effects on poverty and development” [51]. The latter of these two approaches has focused on a group of 13 specific protozoan, helminth, and bacterial infections that have a large global burden of disease and strong poverty-promoting effect, and persist as chronic infections despite effective medical treatments available [53], [54]. (Recently, proposals have expanded this list of NTDs to a total of 17 specific infectious agents [55]). Focusing on the former approach, an important aspect is the direct role of neglect as a contributing factor to NTDs. Previous reviews have described the impact of NTDs on the “bottom billion”, i.e., the portion of the human population living in the most impoverished conditions [56]. Similarly, the “vicious cycle” of interrelatedness between poverty and infectious diseases has been noted by the World Health Organization (WHO) [57].

A major component of the “13 NTDs” is the underlying high burden of disease, both from a morbidity and mortality standpoint, as well as from an economic standpoint. One estimate suggests more than 500,000 deaths annually as a result of these diseases [58]. The burden of EHF and MHF globally is substantially lower (and in comparison to the economic impact of the 13 NTDs [59], the overall economic impact of EHF and MHF would be marginal in comparison). To date, approximately 2,300 total EHF and MHF cases have been recognized [3], [60]. There are some data to suggest this number to be a substantial underestimate. Serosurveys in central Africa have reported the prevalence of reactive antibodies to EBOV in human populations to range from 5% to 15%, implying a much high burden of infection [61]–[63]. Since 1976, in large outbreaks of EHF and MHF, the time from initial cases to outbreak confirmation has typically taken months [35], [64]; thus, it is likely that smaller, brief outbreaks or isolated cases frequently go unrecognized, especially in remote areas. During an intense prospective surveillance program from 1981 to 1985 in the Sud-Ubangi region of northwestern DRC, Jezek et al. identified a total of 21 EHF cases, indicating a possible ongoing occurrence of sporadic EBOV infections in this population [65]. Similarly, during an investigation of MHF in Watsa Zone of northeastern DRC, which involved multiple zoonotic introductions in miners working in gold mines (and some subsequent secondary transmissions) between 1998 and 2000, medical staff reported the disease as a locally recognized clinical entity in miners, occurring as far back as possibly the 1980s [39]. Regardless, the overall burden of disease due to EHF and MHF is clearly dwarfed in comparison to those of the 13 NTDs.

**Figure 1:**
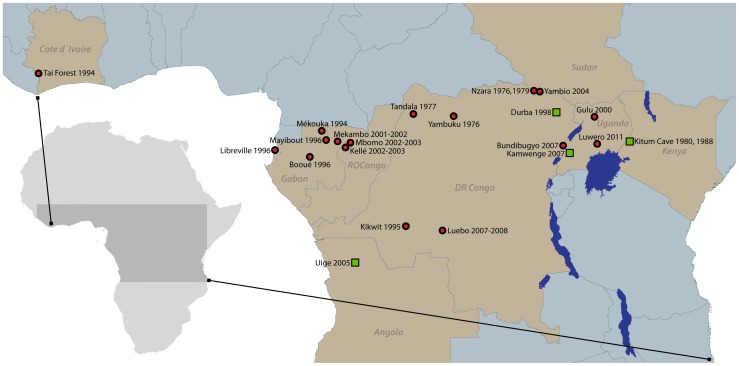
Location of Ebola hemorrhagic fever (red circles) and Marburg hemorrhagic fever (green squares) outbreaks.

In contrast, when EHF and MHF are examined from a bottom billion viewpoint, there are multiple factors supporting the notion that disease, and particularly outbreaks, are components of impoverished conditions. From a geographic standpoint, the bulk of human disease has occurred in rural, and often highly remote, locations in the central African countries of Angola, Gabon, RoC, DRC, Sudan, and Uganda [4] (Figure 1), some of the least developed locations in the world (for instance, see Table 2). As an example of remoteness, 71.3% of the Gulu, Uganda (site of the 2000 EHF outbreak), population live more than 5 km from the nearest health facility, while this percentage is only 0.7% in the capital, Kampala [66]. Although the global distribution of NTDs is more geographically widespread, multiple NTDs also have a high prevalence across this region of Africa [53], [56].

**Table 2:** Select economic and health indicators for countries with previous large outbreaks of Ebola or Marburg hemorrhagic fever (total number of countries which rank is based on).

	Per Capita Income (228^a^)	Infant Mortality per 1,000 Live Births (223^a^)	Life Expectancy in Years, at Birth (222^a^)	Physicians per 1,000 Population (192^a^)
Angola	US$8,200 (121)	175.9 (1)	38.76 (222)	0.08 (169)
Democratic Republic of Congo	US$300 (227)	78.43 (14)	55.33 (199)	0.11 (163)
Gabon	US$14,500 (80)	49.95 (49)	52.49 (207)	0.29 (141)
Republic of Congo	US$4,100 (158)	76.05 (15)	54.91 (200)	0.10 (166)
Sudan	US$2,300 (184)	102.00 (6)^b^	55.42 (198)	0.28 (143)
Uganda	US$1,300 (204)	62.47 (29)	53.24 (204)	0.12 (161)

Data from *The World Factbook*, CIA (accessed December 15, 2011).

a Available number of countries, which rank is based on.

b Data is specific to South Sudan.

Further defining the association between EHF and MHF and impoverished conditions is the observation that amplification of EBOV and MARV transmission commonly occurs in resource-limited health care settings. In addition to transmission associated with re-used medical equipment, many outbreaks involve transmission (sometimes with high frequency) to medical staff caring for patients [24]–[28]. Because EBOV and MARV transmission occurs through direct physical contact with an infected person, bodily fluids, or through contact with contaminated clothes or linens, transmission to health care staff and patients can largely be controlled through implementation of barrier nursing practices for individuals with hemorrhagic symptoms and ensuring that needles or other medical equipment that may contain contaminated fluids are not reused.

Although EHF and MHF may not have the regional or national poverty-promoting effects as some NTDs, the local effects of an outbreak on a village, town, or region can be devastating. Tens to hundreds of deaths have occurred in previous outbreaks. Additionally, these conditions are highly stigmatizing [67]–[69]. Sick patients, medical staff, as well as those who have recovered, commonly face fear and rejection or stigmatization from the local community. Furthermore, the long-term health and psychosocial impacts of EHF and MHF on survivors can be challenging; studies demonstrate post-infection sequelae, as well as prolonged poor health, among those who survived EBOV or MARV infection [69]–[74].

The impact of EHF or MHF on local health systems can be similarly devastating, particularly for individuals in needs of standard medical care not associated with hemorrhagic fever. In the series of Durba-Watsa MHF cases associated with the Durba mine in northwest DRC, the only physician available at Watsa (district) hospital died of presumed MHF in 1994 and no physician was available in the district from 1994 to 1996. A second physician died of MHF in 1999, again leaving the hospital with no available physician. Similarly, the medical director and 11 staff members for a major hospital died of EHF in the Gulu, Uganda, outbreak in 2000 [75], [76]. Beyond the deaths of specific individuals, outbreaks have also had severe effects on the actual functioning of medical services. For instance, the Kikwit, DRC, EHF outbreak in 1995 resulted in the infection of 80 health care workers and the closure of Kikwit General Hospital for non-EHF related activities, severely limiting the availability of medical care to the population of Kikwit (200,000), as well as surrounding areas [26], [29].

An additional defining characteristic of the 13 NTDs is the absence of an available vaccine [77]. Moran et al. previously noted that funding for development of pharmaceutical tools for prevention or treatment is limited for many of the NTDs. For instance, of 2.5 billion US dollars devoted to research and development of new neglected disease products, almost 80% was applied to HIV, tuberculosis, and malaria, with approximately 2% devoted to helminths, and less than 0.1% devoted to Buruli ulcer or trachoma [78]. Regardless, pharmaceutical treatments and cost effective control measures are available for most NTDs [53], [56], underscoring a need for improved implementation of treatment and control efforts. Even in the absence of a vaccine, cases of dracunculiasis (guinea worm disease) have drastically declined through basic public health measures, and guinea-worm eradication is anticipated in the near future [79]. Similarly, no currently licensed vaccines or therapeutics are available for EHF or MHF (discussed further below). While the available funding for research and development of these products may contrast most NTDs, the fact that effective public health measures to prevent or control EHF and MHF are already known is consistent with the above observation for other NTDs.

## Ways Forward

### Improved Surveillance and Health Care Safety

As noted above, a common characteristic of large EHF and MHF outbreaks is the break-down (or absolute lack of) public health surveillance, resulting in long periods of time before identification of the outbreak by public health authorities. With improved surveillance, early chains of transmission can be identified and outbreak response efforts rapidly applied. As an example, during the recent reemergence of EHF in Luwero district, Uganda (May 2011), viral hemorrhagic fever was immediately suspected in the index (and only case) by clinicians at the hospital. While in a rural area, Luwero is located less than 2 hours by vehicle from the capital of Uganda (Kampala). A confirmatory laboratory diagnosis was acquired in less than a week, and outbreak response activities commenced within 24 hours [80]. While contacts of this EHF case fortunately did not develop disease, the ability to identify and follow-up all contacts would have resulted in prevention of further spread of the virus, should secondary cases have developed.

Public health approaches for NTDs have traditionally focused on vertical drug-based treatment strategies [53], [54]. However, the importance of integration of NTD control into broad health systems is now being recognized [81], [82]. Moreover, technical guidelines by the WHO Regional Office for Africa (AFRO) and Member States were recently released for the Integrated Disease Surveillance and Response (IDSR) strategy [83]. The IDSR recommends integrated surveillance of multiple infectious diseases to broaden the ability to detect and respond to infectious diseases of epidemic potential or those targeted for eradication or elimination. Priority diseases included in the 2010 IDSR guidelines include EHF and MHF, as well as a number of other NTDs, including Buruli ulcer, dracunculiasis, leprosy, lymphatic filariasis, onchocerciasis, trachoma, and trypanosomiasis. While public health resources are limited across sub-Saharan Africa, and challenges still exist in its integration, studies have demonstrated tangible improvement of surveillance as a result of IDSR implementation [84].

Laboratory diagnostics are a crucial component of public health surveillance, and efforts need to be made to ensure capacity for rapid diagnostic testing for EHF and MHF across sub-Saharan Africa, as well as the ability to rule out other tropical infections. In the above noted EHF case in Uganda in May 2011, in-country laboratory capacity was available, and a rapid diagnosis was made on the index case, allowing for an immediate public health response [80].

Of additional importance to the control of EHF and MHF is the prevention of health care–associated spread of the viruses. The fact that basic contact precautions (gowns and gloves) can largely block spread of EHF and MHF in health care settings underscores the effect of poverty on the spread of these diseases. Efforts to provide greater availability of basic medical supplies to rural health care settings in central Africa would help minimize the risk of large outbreaks of EHF and MHF, as well as have the broad benefit of preventing non-related health care–associated infections in patients and health care workers, and ensure greater patient safety. A recent report by Marchal et al. stressed potential linkages between NTD control and improvement of health systems [82]. While infection control during medical care is only one aspect of the entire health system, renewed focus on improving health systems may have a direct impact on prevention of initial spread, and ultimately outbreaks, of EHF and MHF.

### Vaccines and Anti-Viral Therapies for EHF and MHF

Extensive research efforts over the past decade have focused on development of vaccines and anti-viral therapies for EHF and MHF, and currently there are numerous promising products in development [85]–[87]. This evidence suggests there may be an optimistic picture for future licensing of efficacious biologic-based measures for EHF and MHF. But are these applicable for those most at risk for disease? There are two scenarios to consider: vaccines that are administered before the exposure, which prevent disease, and vaccines or anti-viral therapies that can be administered after the exposure (either before or possibly after onset of disease) to prevent or improve the clinical prognosis of illness. From an occupation-based risk standpoint, prophylactic vaccination will be clearly a valuable preventive measure, both for individuals with potential exposure in the laboratory or through ecological work, as well as medical and public health personal involved in hands-on outbreak response activities.

When we consider those at risk of endemic exposure to EBOV or MARV—the bottom billion—the potential value of prophylactic vaccination becomes murky. A scenario in which one envisions applying vaccine across the entire endemic population in sub-Saharan Africa is unrealistic. Given the total burden of filovirus disease (∼2,300 total cases identified since 1967), attempting the administration of millions of doses of vaccine has limited justification, particularly considering the current ongoing challenge of establishing high levels of coverage of routine immunizations in many endemic areas. For instance, estimated coverage of polio and measles among 1-year-olds in Uganda in 2009 was 59% and 68%, respectably [88].

A second prophylactic vaccination strategy would be to apply a targeted or mass vaccination campaign to an entire region, in the event of an outbreak. Given the nature of the spread of EBOV and MARV in outbreak settings (chains of person-to-person transmission), the efficacious outbreak control measure already developed (contact tracing, isolation, and safe burials), and the scope of even the largest outbreaks (Gulu, Uganda in 2000 with 425 EHF cases is the largest known outbreak), the application of mass vaccination would not be an efficient or cost effective control mechanism, and would likely draw resources and public personnel away from outbreak control activities. Additionally, if vaccine is administered to an exposed individual during the incubation period and disease subsequently develops, there is a risk that those administering the vaccine will be perceived by the local population as spreading the disease, which would undermine efforts to further implement vaccination or other control methods.

A final strategy, in the instance of a vaccine or anti-viral drug with the potential to prevent or minimize severity of disease, would be to apply these measures to high risk contacts of suspected or confirmed EHF or MHF cases, as well as to those who are already ill (and in isolation). This activity, if measures can be administered early enough to be effective, would inevitably save lives and would be an incentive for suspected patients to enter isolation. However, from an outbreak control standpoint, a symptomatic individual tracked through contact tracing activities is in essence removed from the “transmitter pool”, and shortly after onset of symptoms (and infectiousness) will be placed under safe isolation for proper medical care. Similarly, sick individuals, already in properly managed isolation, would not further propagate the virus. Thus, while having potential therapeutic value, post-exposure biologics may have limited impact on the scope of EHF or MHF outbreaks.

Finally, it worth reiterating in the broad context of vaccines or anti-viral therapies for outbreak settings, that any application is contingent on identification of the outbreak. Since traditionally in large outbreaks, a high proportion of cases occur before outbreak identification, biologic-based prevention measures would have no impact on these cases. With effective surveillance, initial cases can be identified rapidly, minimizing the overall impact of the outbreak through classic outbreak control measures. Thus, while not a stand-alone intervention for outbreak control, application of anti-viral therapies may help lower the overall impact of fatalities in EHF and MHF outbreaks.

## Conclusions

Those most at risk for EHF and MHF are residents of rural central Africa, many of whom are among the bottom billion. Outbreaks of EHF and MHF are commonly associated with limited public health surveillance and inadequate medical preventive measures, both partially the result of impoverished conditions. Effective methods to prevent and control EHF and MHF are well understood. While challenging, efforts to combine control of these diseases with other NTDs, through mechanisms such as integrated surveillance and improvement of health systems, would provide a combined benefit to populations in rural central Africa. While multiple candidate vaccines and anti-viral therapies against EBOV and MARV are currently in development, classical public health surveillance and outbreak control guidelines will likely remain the cornerstone of disease control. However, modern therapies have the potential to minimize the number of EHF and MHF deaths in outbreak settings.

**Key Learning Points**

- Ebola hemorrhagic fever (EHF) and Marburg hemorrhagic fever (MHF) cause outbreaks with high case fatality in central Africa.
- The overall incidence of EHF and MHF is low; however, outbreaks can have devastating local and regional consequences. EHF and MHF outbreaks are facilitated by impoverished conditions, where available public health and safe medical facilities are limited.
- Integration of EHF and MHF surveillance and response into public health systems for common NTDs may help in the control of both sets of diseases.
- Although vaccines may not prevent all future outbreaks, there are promising vaccines for EHF and MHF on the horizon.

**Key Papers**

- Anonymous (1978) Ebola haemorrhagic fever in Zaire, 1976. Bull World Health Organ 56: 271–293.
- Anonymous (1978) Ebola haemorrhagic fever in Sudan, 1976. Report of a WHO/International Study Team. Bull World Health Organ 56: 247–270.
- Khan AS, Tshioko FK, Heymann DL, Le Guenno B, Nabeth P, et al. (1999) The reemergence of Ebola hemorrhagic fever, Democratic Republic of the Congo, 1995. Commission de Lutte contre les Epidemies a Kikwit. J Infect Dis 179 Suppl 1: S76–S86.
- Hotez PJ, Molyneux DH, Fenwick A, Kumaresan J, Sachs SE, et al. (2007) Control of neglected tropical diseases. N Engl J Med 357: 1018–1027.
- Liese B, Rosenberg M, Schratz A (2010) Programmes, partnerships, and governance for elimination and control of neglected tropical diseases. Lancet 375: 67–76.
